# Transport mechanism of a glutamate transporter homologue Glt_Ph_

**DOI:** 10.1042/BST20160055

**Published:** 2016-06-09

**Authors:** Yurui Ji, Vincent L.G. Postis, Yingying Wang, Mark Bartlam, Adrian Goldman

**Affiliations:** * College of Environmental Science and Engineering, Nankai University, Tianjin 300071, China; † Astbury Centre for Structural Molecular Biology, School of Biomedical Sciences, University of Leeds, Leeds, LS2 9JT, U.K.; ‡ Biomedicine Research Group, Faculty of Health and Social Sciences, Leeds Beckett University, Leeds, LS1 3JT, U.K.; § College of Life Sciences, Nankai University, Tianjin 300071, China; ║ Division of Biochemistry, Department of Biosciences, University of Helsinki, Helsinki, FIN-00014, Finland

**Keywords:** aspartate transporter, Cl^−^ conductance, excitatory amino acid transporters (EAATs), Glt_Ph_, glutamate transporter, Na^+^ coupling

## Abstract

Glutamate transporters are responsible for uptake of the neurotransmitter glutamate in mammalian central nervous systems. Their archaeal homologue Glt_Ph_, an aspartate transporter isolated from *Pyrococcus horikoshii*, has been the focus of extensive studies through crystallography, MD simulations and single-molecule FRET (smFRET). Here, we summarize the recent research progress on Glt_Ph_, in the hope of gaining some insights into the transport mechanism of this aspartate transporter.

## Introduction

Glutamate transporters, also known as excitatory amino acid transporters (EAATs), belong to the dicarboxylate/amino acid:cation (Na^+^ or H^+^) symporter (DAACS) family [1]. In the mammalian central nervous system, neuronal and glial EAATs transport glutamate, the main neurotransmitter, from the outside to the inside of the nerve cells, removing excessive excitotoxic glutamate, which may cause neurotoxicity [2,3]. Various human diseases, such as Alzheimer's disease, epilepsy and strokes, have been linked to dysfunction of EAATs [4,5].

In humans, there are five subtypes of glutamate transporters (EAAT1–5) [6]. The transport of glutamate is driven by energy derived from ion gradients, mostly Na^+^ [2,3,6]. In EAATs, three Na^+^ ions and one proton are co-transported with glutamate and the transport cycle is completed by the counter-transport of one K^+^ ion [7]. In addition to the ion-coupled transport, EAATs also display uncoupled chloride conductance [8–11] and have different preferences towards ions [10]. Therefore, glutamate transporters function both as secondary active transporters and anion-selective ion channels [8,10,12].

Despite the importance of glutamate transporters in mammalian systems, there are currently no crystal structures of a mammalian EAAT. One archaeal homologue of the glutamate transporter, Glt_Ph_, isolated from *Pyrococcus horikoshii* glutamate transporter, has however been extensively studied over the past ten years. It shares 37% sequence identity with human EAAT2 [13,14] and many functionally important amino acid residues are highly conserved between Glt_Ph_ and its human homologues [13], making it an excellent model system for researchers to use.

Glt_Ph_ transports aspartate together with three Na^+^ ions into the cytoplasm [15], accompanied by a stoichiometrically uncoupled Cl^−^ conductance as well [16]. There are thus three major differences between it and the human EAATs: first that no proton is symported with aspartate [17], second that K^+^ ion counter-transport is not required to complete the transport cycle [17] and third, a strong preference for aspartate over glutamate [18]. In contrast, EAATs require one proton for co-transport [7], one K^+^ ion counter-transport to complete the transport cycle [7] and transport glutamate and aspartate with similar affinity [8,11,19]. In this review, we summarize the current state of structural studies, MD simulations and single-molecule FRET (smFRET) studies of Glt_Ph_ that have provided insights into its transport mechanism–and by extension, the mechanism of the EAATs as well.

## Overall structure and domain motions of Glt_Ph_

**Figure 1:**
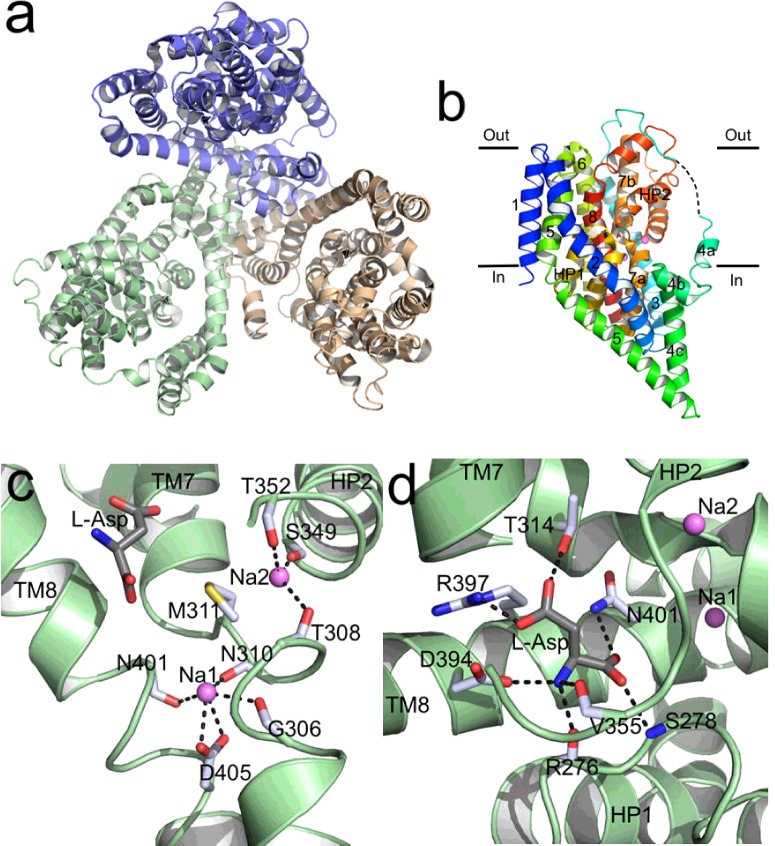
Structures of Glt_Ph_ (**a**) Trimer of Glt_Ph_ viewed from the extracellular side in the outward-facing state. Each monomer, in cartoon, is coloured differently. (**b**) Cartoon representation of a monomer of Glt_Ph_ shown parallel to the membrane in the outward-facing state with aspartate and two Na^+^ ions bound. The TM helices and re-entrant loops are labelled. The substrate is shown as stick and the two Na^+^ ions are shown as purple spheres. The black dashed lines between TM3 and TM4a represent the loop connecting the helices. (**c**) View of the Na^+^-binding sites. (**d**) View of the aspartate-binding site. Dashed lines show the hydrogen bonds between amino acid residues and Na^+^ or aspartate.

The outward-facing state, captured in the first crystal structure of Glt_Ph_ [13], revealed a homotrimer (Figure 1a) with a bowl-shaped extracellular-facing basin whose surface is hydrophilic and as deep as half of the trimer's height. Each wedge-shaped protomer (Figure 1b) consists of two domains: a trimerization domain formed by four transmembrane (TM) helices (TM1, TM2, TM4 and TM5) providing interactions between subunits in the trimer; and a transport domain formed by four TM helices (TM3, TM6, TM7 and TM8) and two re-entrant loops [helical hairpin (HP) structures, HP1–2] [13,20].

Comparison of the aspartate-bound structure and the structure with the competitive inhibitor DL-threo-β-benzyloxyaspartate (TBOA)-bound shows that HP2 serves as the extracellular gate [18]. Glt_Ph_ can adopt an ‘open’ conformation (solved with TBOA bound), which allows substrate access from the outside to its binding site, at which point it switches to the ‘closed’ conformation (solve with aspartate bound). This role of HP2 has also been verified by MD studies [21,22]. HP1 was therefore proposed to function as the intracellular gate as its movement is involved in the dissociation and release into the cytoplasm of the substrate and ions [20]. However, this remains the subject of some controversy in recent MD studies, as will be discussed below (Transport Mechanism).

As the substrate-binding site in both the aspartate- and TBOA-bound structures is approximately 5 Å (1 Å=0.1 nm) beneath the extracellular surface, these two structures are called [20] the outward-facing closed (or occluded) state and outward-facing open state respectively. The inward-facing state is obtained by cross-linking of a double-cysteine mutant introduced into Glt_Ph_ [20] (Table 1). For example in the structure of Glt_Ph_-K55C–A364C_Hg_, aspartate is bound approximately 5 Å beneath the intracellular surface [20].

**Table 1:** Summary of all currently-available crystal structures of Glt_Ph_

PDB ID code	Inward- or Outward-facing	Description
1XFH	Outward-facing	7 histidine introduced and Glt_Ph_7H used for crystallization
2NWL	Outward-facing	Glt_Ph_7H with aspartate
2NWW	Outward-facing	Glt_Ph_7H with TBOA
2NWX	Outward-facing	Glt_Ph_7H with aspartate and Na^+^
3V8G	Intermediate outward-facing	Cross-linked Glt_Ph_7H V198C–A380C_Hg_ with aspartate and Na^+^. One protomer in intermediate outward-facing state
3V8F	Inward-facing	Cross-linked Glt_Ph_7H V216C–M385C_Hg_ with aspartate and Na^+^
3KBC	Inward-facing	Cross-linked Glt_Ph_7H K55C–A364C_Hg_ with aspartate and Na^+^
4IZM	Outward-facing	Cross-linked Glt_Ph_7H L66C–S300C_Hg_ with aspartate and Na^+^
4P1A	Inward-facing	Cross-linked Glt_Ph_7H K55C–A364C_Hg_ with thallium bound (apo conformation)
4P19	Inward-facing	Apo cross-linked Glt_Ph_7H K55C–A364C_Hg_
4P3J	Inward-facing	Apo cross-linked Glt_Ph_7H K55C–A364C_Hg_ in alkali-free conditions
4P6H	Inward-facing	Cross-linked Glt_Ph_7H K55C–A364C_Hg_ with thallium bound (bound conformation)
4OYE	Outward-facing	Glt_Ph_6H R397A with no ligands bound
4OYF	Outward-facing	Glt_Ph_ R397A with Na^+^ bound
4OYG	Outward-facing	Glt_Ph_7H R397A with aspartate and Na^+^
4X2S	Inward-facing	Glt_Ph_7H R276S–M395R with aspartate and Na^+^

Biochemical, crystallographic and double electron–electron spin resonance [DEER (also called PELDOR)] spectroscopy data all demonstrate that the trimerization domain serves as a scaffold and stays in almost the same conformation during ligand binding and transport [20,23,24], whereas the transport domain, stabilized by the scaffold, undergoes large conformational changes involving a TM translation and rotation [20]. Various studies with different techniques performed on EAATs show that individual subunits in the homotrimer function independently [25–28]. Although there is no direct evidence about how the subunits in Glt_Ph_ function, it should be similar to the EEATs, given the high level of similarity between Glt_Ph_ and the EAATs.

Rigid body movement (called ‘elevator-like’ motions [29]) of the transport domain can be observed when comparing the structures of apo or holo outward-facing and inward-facing Glt_Ph_ respectively [20,30]. The elevator-like motions of the transport domain have also been observed in a smFRET study of the wild-type and a humanized mutant (R276S/ M395R) of Glt_Ph_ [31], suggesting that these motions mediate substrate uptake and are pivotal steps of the transport cycle [31,32].

Both DEER [24] and smFRET [32,33] studies on Glt_Ph_ show that the protomers in the trimer can sample different conformations randomly and independently, and individual transport domains alternate between periods of quiescence and periods of rapid transition. This is also captured in the Glt_Ph_-V198C–A380C_Hg_ crystal structure, with one of the protomers in the intermediate outward-facing state and the other two in the inward-facing state [34].

## Na^+^ ion binding

The positions of two Na^+^ ions (Na1 and Na2) have been experimentally identified: there is no direct interaction between these two Na^+^ ions and the bound aspartate [18]. In the outward-facing holo crystal structure, Na1 is located below the aspartate, coordinated by the main chain carbonyls of Gly^306^ and Asn^310^ (TM7), of Asn^401^ (TM8) and the Asp^405^ side chain (TM8) (Figure 1c). Of these residues, Asp^405^ is the most important: it coordinates Na1 bidentately via the γ-carboxylate group, and analysis of data from the Glt_Ph_–D405N crystals soaked in Tl^+^ solution (an Na^+^ mimic) found a strong peak only at the Na2, not the Na1, position and the mutant bound aspartate more weakly [18]. In the outward-facing holo crystal structure, Na2 is below the re-entrant helical HP2, coordinated by the carbonyl groups of Thr^308^ and Met^311^ (TM7) and of Ser^349^ and Thr^352^ (HP2) [18] (Figure 1c).

In both the outward-facing and inward-facing holo crystal structures, the distance between the hydroxy group of Thr^308^ side chain and the backbone carbonyl of Pro^304^ is approximately 4.8 Å, which is too far to form a hydrogen bond. This allows Thr^308^ to coordinate the Na^+^ ion at Na2. However, the Pro^304^–Thr^308^ hydrogen bond exists in the outward-facing apo crystal structure [30] and the outward-facing crystal structure of Glt_Ph_ with TBOA bound [18]. In the outward-facing apo crystal structure, the HP2 loop is collapsed into the aspartate binding and Na2 sites as well [30]. In the structure of Glt_Ph_ in complex with TBOA, HP2 moves approximately 10 Å away from the position where it is in the outward-facing holo structure and therefore cannot coordinate an Na^+^ ion at Na2 [18]. Steered molecular dynamics (SMD) simulations suggested that the breaking of the hydrogen bond between Pro^304^ and Thr^308^ destabilizes the last turn of the TM7a helix and allows readjustment of the backbone carbonyl oxygen atoms, placing them in a favourable position to coordinate the second Na^+^ ion [35]: this role for Thr^308^ has been verified by experimentally measuring the involvement of three Thr^308^ mutants (T308W/T308A/T308V) in binding and transport [35]. However, superposition of the outward-facing apo Glt_Ph_ structure and the outward-facing Glt_Ph_ structure with Na^+^ bound at Na1 shows that ion binding to Na1 releases HP2 to free the aspartate binding and Na2 sites into a conformation similar to that in the outward-facing holo structure, breaking the hydrogen bond between Thr^308^ and Pro^304^ as well [30].

The third Na^+^-binding site (Na3) is difficult to observe structurally, because binding at the third site would lead to conformational change and transport. Consequently, opinions vary regarding its position [36–38]. An MD simulation [38] based on the aspartate-bound and TBOA-bound structures [18] predicted a new binding site for Na3, which differs from previous MD simulation results [36,37]. Bastug et al. [38] predicted that the third Na^+^ ion is coordinated by the side chains of Thr^92^, Ser^93^, Asn^310^, Asp^312^ and the backbone of Tyr^89^. They were able to verify this experimentally: the T92A and S93A variants showed different changes in aspartate affinity but both exhibited a reduction in Na^+^ affinity compared with wild-type Glt_Ph_. In addition, Asn^310^ and Asp^312^ are both part of the highly conserved NMDGT motif [18]; Thr^314^ in the motif is involved in aspartate binding [18] and mutations of the equivalent residue (Thr^400^) in EAAT2 abolish its function [39].

## Uncoupled chloride ion conductance

A stoichiometrically uncoupled Cl^−^ conductance is observed along with aspartate transport in Glt_Ph_ [16]. This Cl^−^ conductance can partially neutralize the membrane potential caused by the electrogenic substrate transport. The anion selectivity of Glt_Ph_ is almost the same as that of EAATs. Mutation of a conserved amino acid (S65V in Glt_Ph_, located in TM2) strongly affects the chloride conductance with almost no effect on the Na^+^: aspartate symporter [16], similar to results observed in EAAT1 (S103V) [40]. Clearly, Cl^−^ permeates through a specific pathway [16] and Ser^65^ is somehow involved in the process. In a recent MD simulation [41], however, researchers were unable to find any evidence showing that Ser^65^ interacts directly with Cl^−^. Combined with experimental evidence obtained from both Glt_Ph_ and EAAT4, they proposed that Ser^65^ exerts its effect on anion permeation by altering the rates of conformational changes leading to the open anion channel.

A recent study combined MD simulations with fluorescence spectroscopy of Glt_Ph_ and patch-clamp recordings of mammalian EAATs [41]. The authors suggested that lateral movement of the transport domain triggers formation of the anion-selective permeation pathway only if the domain sampled intermediate transporter conformations, rather than outward- or inward-facing states. They predicted residues that line the ion permeation pathway by simulation and verified these predictions through fluorescence spectroscopy and functional studies on mutant transporters. Of the residues lining the pathway, the side chain of Arg^276^ protrudes from the tip of HP1 into the Cl^−^ permeation pathway and this resulting positive charge contributes to the anion selectivity for both Glt_Ph_ and the EAATs [41]. This residue is also involved in the binding of substrates [18,30]. Interaction with the substrate does not compromise its role in anion permeation and selectivity [41].

## Substrate affinity and binding

Although Glt_Ph_ is a glutamate transporter homologue, it exhibits a strong preference for aspartate as a substrate in the presence of an Na^+^ gradient. It shows 60000-fold higher affinity for aspartate (with *K*_d_ values for aspartate and glutamate of approximately 2 nM and 122 μM respectively) [18]. The aspartate-binding site consists of the tips of HP1 and HP2, the conserved NMDGT motif of TM7 (see above) and hydrophilic residues on TM8 [18] (Figure 1d). The α-carboxyl group of the substrate interacts with the side chain of Asn^401^ (TM8) and the main chain amide nitrogen of Ser^278^ (HP1), whereas the γ-carboxyl group interacts with the side chains of Thr^314^ (TM7) and Arg^397^ (TM8). The substrate amino group interacts with the side chain of Asp^394^ (TM8) and the backbone carbonyl groups of Arg^276^ (HP1) and Val^355^ (HP2) (Figure 1d).

## Transport mechanism of the aspartate transporter Glt_Ph_

Binding thermodynamics studies show that aspartate binding and release, rather than TM movements of the transport domains, is coupled to the chemical potential of sodium ions in solution [42].

Structural comparison of outward-facing apo and holo-Glt_Ph_ shows that in the apo structure, there is joint movement of HP2 and TM8a and also reorganization of ligand-binding sites including HP2, the NMDGT motif and TM3. The HP2 loop region collapses into the substrate- and Na2-binding sites. The movements of side chains in the NMDGT motif (Asn^310^ and Met^311^) and the bending away of TM3 from the motif deform the Na1 site [30]. These distortions mean than Na^+^ can no longer bind. (Similar distortion of ligand-binding sites also has been observed in the outward-facing apo structure of Glt_Tk_ [43], which has 77% sequence identity with Glt_Ph_).

**Figure 2:**
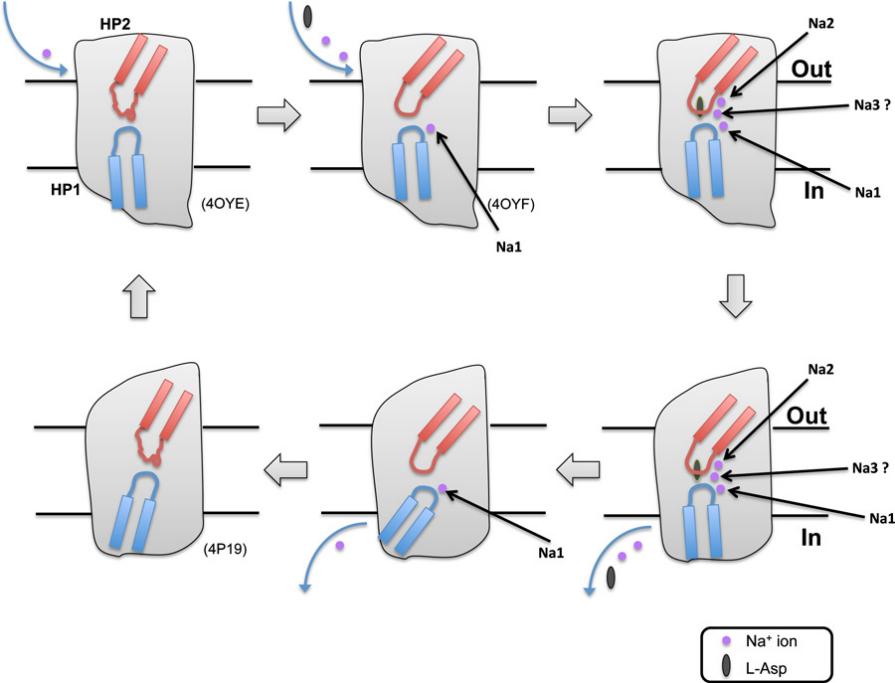
The Glt_Ph_ transport cycle Model of the Glt_Ph_ transport cycle for a monomer based on available crystal structures and MD simulations on the binding and release order of the ligands. Protein data bank (PDB) codes are in parentheses. The helical HP structure in red is HP2 and the blue one is HP1. The purple circles represent Na^+^ ions binding at Na1, Na2 and Na3. The grey ellipse represents aspartate. Starting from the upper left corner, in the outward-facing apo structure, Na^+^ ion binding at Na1 triggers structural changes in the transport domain and HP2, which opens the aspartate and Na2 sites to conformations similar to that in the holo transporter [30]. After aspartate and Na^+^ ion bind to their corresponding binding sites, there is a further, unknown conformational change linked to the binding of Na3 before movement across the membrane. Once the transport domain reaches the intracellular side, through opening of the intercellular gate, the substrates release into cytoplasm. The transport domain stays compacted with collapsed ligand-binding sites, which make it suitable for TM movement, thus completing the transport cycle [30]. There are as yet no experimental data on the position of the third Na^+^ ion-binding site or the binding order of the ligands.

Binding of Na^+^ and aspartate trigger different movements of HP2, with the binding of the former causing HP2 to open and allow binding of Na2, whereas the binding of the latter causes HP2 to close [44]. Binding of aspartate and the Na^+^ at the Na2 site is coupled as both sites are partly formed by the tip of HP2 [30] (Figure 2). A binding thermodynamics study of Glt_Ph_ also suggests that binding of the first two Na^+^ is involved in the modification of the substrate-binding site, whereas the binding of the third Na^+^ is coupled to the substrate occlusion from outside solvent [42]. During the ligand binding process, with the exception of extracellular gate HP2 closure, other unknown conformational changes dominate the process and remain to be elucidated by further research [42]. After the ligands are fully bound to the transport domain and occluded from the solvent by the closure of both HP1 and HP2, the transport domain moves across the membrane as a rigid body [20] (Figure 2).

Simulations based on the inward-facing crystal structure of Glt_Ph_ have provided preliminary insights into the process of substrate release into the cytoplasm. DeChancie et al. [45] suggested that release is initiated by dissociation of Na^+^ from the Na2 site and, almost simultaneously, opening of the HP2 loop exposes the substrate and other polar and charged groups. This attracts water molecules to the substrate-binding site, which further destabilizes interactions between substrate and protein residues on HP2 and TM8. The HP1 loop then opens, disrupting the strong hydrogen bonds between the SSS motif (Ser^277^–Ser^279^) on the HP1 loop and the substrate, allowing the aspartate to dissociate. In this model, HP2 serves as an activator of the intracellular HP1 gate [45]. However, a previous simulation suggested that HP2 is in fact the intracellular gate in the inward-facing state [46]. In this model, HP2 opening is a prerequisite for substrate release into the cytoplasm. Understanding the mechanism of substrate release requires further research.

Following substrate release, the transport domain undergoes a series of conformational changes to prepare itself for the TM movement. The conformational changes in the inward-facing apo structure are that though all of the ligand-binding sites are distorted, the apo transport domain is as closed and compact as in the fully bound structure [30] (Figure 2). This may be critical for the transport domain to transit to the outward-facing state.

## Outlook

Although crystallographic, MD simulations and smFRET studies have greatly increased our understanding of the Glt_Ph_ transport mechanism, there are still many questions yet to be answered, including a definitive answer to the position of the third Na^+^ ion, the mechanism of substrate binding and release, and how the transport cycle is completed. Single-molecule and structural studies backed up by computational studies should yield definitive insights into the mechanism of substrate release and the transition to the outward-facing state in Glt_Ph_. However, to understand the differences between it and the EEATs, for instance the differing substrate and ion transport specificity, will require high-resolution structures of the EEATs, either by X-ray crystallography or–possibly–by EM using the new generation of microscopes.

## Abbreviations

DEER: double electron–electron spin resonance
EAAT: excitatory amino acid transporter
Glt_Ph_: *Pyrococcus horikoshii* glutamate transporter
HP: hairpin
smFRET: single-molecule FRET
PDB: protein data bank
TBOA: DL-threo-β-benzyloxyaspartate
TM: transmembrane
